# Recommendations for avoiding knee pain after intramedullary nailing of tibial shaft fractures

**DOI:** 10.1186/1754-9493-5-31

**Published:** 2011-12-01

**Authors:** Pedro José Labronici, Robinson Esteves Santos Pires, José Sérgio Franco, Hélio Jorge Alvachian Fernandes, Fernando Baldy dos Reis

**Affiliations:** 1 Department of Orthopaedic Surgery, Santa Tereza Hospital, Petrópolis, RJ, Brazil; 2 Department of Orthopaedic Surgery, Federal University of Minas Gerais and Felício Rocho Hospital, Belo Horizonte, MG, Brazil; 3 Department of Orthopaedic Surgery, Federal University of Rio de Janeiro, Rio de Janeiro, RJ, Brazil; 4 Department of Orthopaedic Surgery, Federal University of São Paulo, São Paulo, SP, Brazil

## Abstract

**Background:**

The objective of this study is to analyze the proximal tibiofibular joint in patients with knee pain after treatment of tibial shaft fractures with locked intramedullary nail.

**Findings:**

The proximal tibiofibular joint was analyzed in 30 patients, who reported knee pain after tibial nailing, and standard radiograph and computed tomography were performed to examine the proximal third of the tibia. Twenty patients (68.9%) presented the proximal screw crossing the proximal tibiofibular joint and 13 (44.8%) had already removed the nail and/or screw. Four patients (13.7%) reported complaint of knee pain. However, the screw did not reach the proximal tibiofibular joint. Five patients (17.2%) complained of knee pain although the screw toward the joint did not affect the proximal tibiofibular joint.

**Conclusion:**

When using nails with oblique proximal lock, surgeons should be careful not to cause injury in the proximal tibiofibular joint, what may be one of the causes of knee pain. Thus, the authors suggest postoperative evaluation performing computed tomography when there is complaint of pain.

## Background

Tibial shaft fracture is considered the most common long bone in orthopaedic practice. Fixation with intramedullary nail has frequently been used and proven to be efficient in displaced tibial shaft fractures [1-8]. Tibial nailing is related with relatively low incidence of nonunion, malunion, infection and compartmental syndrome [9-11]. However, pain in the knee joint is the most common complication after tibial nailing. Its occurrence has been reported from 10 to 86% of the cases, particularly in young and active patients [12-16]. A recent meta-analysis of the literature has estimated an incidence of 47.4% [11]. Although the etiology of the knee pain after intramedullary nail is still unknown, many theories have been proposed [11,13,17-19].

Several anatomic structures around the knee are prone to damage during nail insertion, including the patellar tendon [11,14], menisci, articular cartilage, the infrapatellar branch of the saphenous nerve and infrapatellar fat pad [14]. Additionally, the presence of prominent nail and/or screw and the associated muscular weakness have been described as causative factors of pain [20]. Some nail designs, with the oblique screw in the proximal aspect, have proven to be biomechanically more stable in tibial fractures. Nevertheless, with this type of fixation, the proximal screw may injure the proximal tibiofibular joint, what may cause knee pain [21].

Our purpose was to analyze the proximal tibiofibular joint, performing computed tomography in 30 patients with knee pain after locked intramedullary nailing in tibial shaft fractures.

## Methods

We retrospectively reviewed a hundred patients with displaced tibial shaft fractures treated with locked intramedullary nailing in a general hospital, between 2000 and 2004. Thirty patients reported knee pain after the procedure and were analyzed through standard radiograph and computed tomography of the proximal third of the tibia (axial cuts). All surgeries were performed by the same surgeon utilizing the same nail design. A patient lost the computed tomography and was excluded. All patients had been treated with unreamed locked intramedullary nail with proximal oblique screws. The age ranged from 18 to 71 years old. The mean age was 36 years old. Twenty-two were male and seven were female.

The distance between the nail with the tibial plateau and the anterior cortex of the tibia was analyzed in the lateral view radiographs according to the method of Keating et al [13]. All radiographs were taken with standard distance of 90 cm. The height of the nail was defined in the lateral view radiograph as the distance between the line drawn through the tibial plateau and a parallel line to this strip that touches the apex of the nail. Negative values mean that the nail would be buried in the proximal metaphyseal aspect of the tibia. Positive values show the amount of prominence of the nail in relation to the tibial plateau. The distance of the cortex with anterior nail was defined between a line drawn above the anterior cortex of tibia and the anterior apex of the nail. Patients without nails and/or screws were included, assessing the measures from previous radiographs.

**Figure 1:**
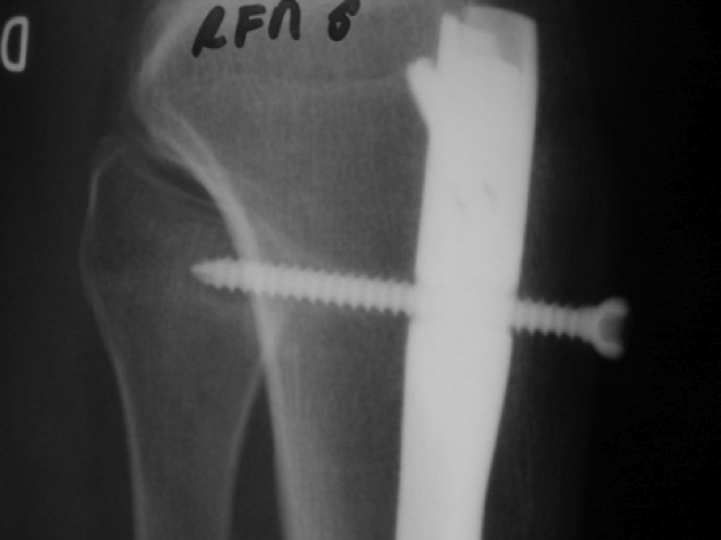
**X-ray in lateral view with the oblique screw crossing the proximal tibiofibular joint**.

**Figure 2:**
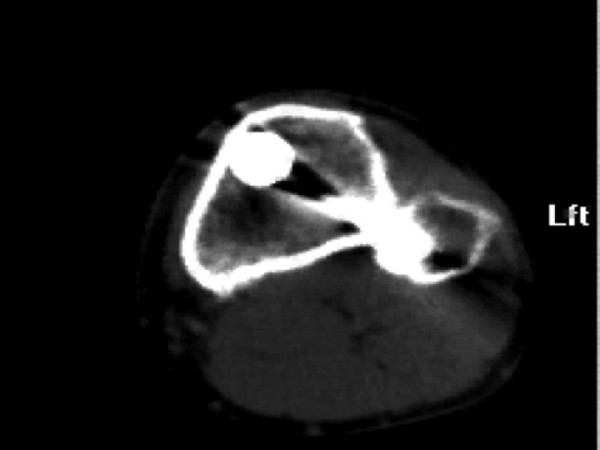
**Computed tomography in axial cut showing the screw crossing the proximal tibiofibular joint**.

The CT scan of the proximal metaphyseal aspect of the tibia was evaluated for the proximal locking screw crossing the proximal tibiofibular joint, avoiding the joint or directed toward the joint but without penetrating the joint. (Figures 1 and 2).

Approval by the ethics committee of the institution involved in the study was obtained.

## Results

Among a hundred patients treated with intramedullary nailing after tibial shaft fractures, 30% complained of knee pain. A patient lost the computed tomography and was excluded. Performing the computed tomography, it was observed that 20 patients (68.9%) had the proximal screw crossing the proximal tibiofibular joint and 13 (44.8%) had already taken out nail and/or screw. Four patients (13.7%) presented complaints of knee pain although the screw did not reach the proximal tibiofibular joint and there was no nail prominence in tibial plateau level as well as in the anterior cortex of tibia. In five patients (17.2%), the screw was toward the joint, but it did not affect the proximal tibiofibular joint. In one patient, the nail was 0.5 mm prominent in relation to the anterior cortex of tibia and another presented the nail 0.5 mm prominent in relation to the anterior cortex and 0.5 mm in the tibial plateau level. Four patients (13.3%) presented the nail above the tibial plateau varying from 0.3 mm to 0.7 mm with an average of 0.5 mm. Of these, three also presented the screw in the proximal tibiofibular joint. In six patients (20.6%), the prominent nail in relation to the anterior cortex of tibia ranged between 0.3 mm and 0.5 mm with an average of 0.4 mm. Four of these had the screw in the proximal tibiofibular joint (Table 1).

**Table 1:** Patients with complaint of knee pain after intramedullary nailing.

Age	Gender	Screw in the joint	Screw out of the joint	Without nail and/or screw	Screw toward the joint	Nail above the plateau	Prominent nail anterior cortex
57	FEMALE	X				NO	NO
57	MALE				X	0,5 mm	0,5 mm
25	MALE	X		X		NO	NO
33	MALE	X		X		NO	NO
29	FEMALE	X		X		NO	NO
43	FEMALE	X		X		NO	NO
26	FEMALE	X		X		NO	NO
31	MALE	X				NO	0,3 mm
18	MALE	X		X		NO	NO
19	MALE	X				NO	NO
30	MALE	X				NO	NO
41	MALE		X			NO	NO
52	MALE				X	NO	0,5 mm
56	FEMALE		X			NO	NO
71	FEMALE		X			NO	NO
27	MALE	X				NO	NO
38	MALE	X		X		NO	0,4 mm
54	MALE			X	X	NO	NO
29	MALE	X				NO	NO
59	MALE	X		X		0,3 mm	0,3 mm
36	MALE	X				0,6 mm	0,3 mm
31	MALE				X	NO	NO
36	FEMALE	X				NO	NO
21	MALE	X				0,7 mm	NO
21	MALE	X		X		NO	NO
18	MALE			X	X	NO	NO
26	MALE	X		X		NO	NO
27	MALE	X		X		NO	NO
34	MALE		X			NO	NO

## Discussion

There are many studies describing knee pain after treatment of tibial shaft fractures with locked intramedullary nail [11,12,16,18,21-23].

Although the etiology of pain is still unknown, it seems to be multifactorial. Several studies have been carried out to identify the causes of pain [11,13,14,20]. Meta-analysis with 20 studies evaluating knee pain after intramedullary nail has shown a mean prevalence of 47.4% (10% - 86%) [11]. This study has shown prevalence of 30%, which could be attributed to the fact that we used unreamed nail, with smaller diameter, which consequently caused less injury to the bone and to the soft tissues during the nail insertion.

The choice of surgical approach, particularly through the transpatellar or parapatellar tendon, has been reported as a contributory factor of knee pain after nail insertion. Several authors suggest that the transpatellar approach is more likely to present higher risks. Furthermore, it is more commonly associated with knee pain in the post-operatory due to its incision through the tendon, to the retro-tendinous fat pad-injury, which is highly innervated [13,17,24,25]. When parapatellar approach is used, the patellar tendon, the retropatellar fat pad and the tissues are retracted and, theoretically there would be no tissue injury. The trauma caused by the retractors during reaming could cause knee pain [11,13,14,19,26]. In this study, we used the medial parapatellar approach for nail insertion in all patients. It was our belief that with this approach we would be able to protect the tendon from one more trauma decreasing the likelihood of causing knee pain.

The distance of the nail, from the proximal entry point in the tibial plateau or in the anterior tibial cortex, does not have relation with knee pain [27]. Keating et al [13] reported that the mean distance between the nail and the plateau was 13 mm and concluded that knee pain happened independent of this distance. Additionally, knee pain was observed in 57% of the patients with 5 mm of the nail in the exterior aspect of the anterior cortex of tibia. Utilizing the distance nail-plateau and nail-anterior cortex as parameters of assessment, Bhattacharyya et al [15] demonstrated that, when the nail was buried 1.25 cm in relation to the tibial plateau, in the coronal and sagittal axes, the pain decreased significantly. However, the authors did not establish a limit [15]. Uzumcugiletal et al [27] verified that in the knee pain group, although the mean distance in the tibia plateau was 11.5 ± 7.9 mm, the pain persisted. In a meta-analysis published in 2006, which examined knee pain after intramedullary nailing procedure, it was suggested that the prominent nail should be avoided. However, no absolute values of de distance were shown [11]. Our study observed that 13.3% of the patients with knee pain had the nail above the tibial plateau. In three, pain was related to the screw in the proximal tibiofibular joint. In six patients, (20.6%) the nail was prominent to the anterior tibial cortex. Of these, in four the pain was associated with the screw in the proximal tibiofibular joint. Nevertheless, it is important to point out that the patients who did not report knee pain, although they had prominent nail in the tibial plateau and in the anterior cortex aspect, were not assessed. Neither was analyzed the depth of the nail in relation to the proximal interlock. It is also important to highlight that, when using some nails with oblique lock, it is necessary to cross the lateral cortex to obtain fracture stability.

Laidlaw et al [21] were the first to document the injury in the proximal tibiofibular joint after insertion of the medial interlock screw with oblique lock nails in tibial shaft fractures. They used the interface of a clock to a correlated to a lock in the position of 2 o'clock, as intra-operatory evaluation of the proximal tibiofibular joint. Thus, they avoided the "danger zone", which was found to be between 44.7° to 72.1° on the right from 40.6° to 73.0° on the left. They also pointed out that the surgeons should be aware of this complication and avoid either the placement of the screw in the posterolateral direction or the interlock screw crossing the lateral cortex. In our study, 30 patients complained of knee pain when treated with proximal oblique interlock nails. Of these, 20 presented injury in the joint, confirmed through computed tomography. We believe that, when using this nail design, the surgeon should be careful with the direction of the interlock screw to avoid injury in the tibiofibular joint. Thus, preventing one more causative factor after placement of intramedullary nail in tibial shaft fractures.

As strengths, this study demonstrates that the direction of the proximal screw from anteromedial to posterolateral, depending on the height of the nail can reach the proximal tibiofibular joint and be a potential cause of knee pain. Current literature is scarce on the subject and we believe that this information will serve as a warning to orthopaedic surgeons about this potential complication. In addition, the first study that cited such complications had a sample size of 2 patients [21]. This study presents 20 patients with violation of the proximal tibiofibular joint with oblique locking screws. As weaknesses of this paper, we address specifically a potential cause of knee pain after osteosynthesis of tibial shaft fractures with locked intramedullary nails. We can't attribute the pain exclusively to this cause, since knee pain after tibial nailing is multifactorial.

As a final comment, it should be noted, as Bhattacharyya et al [15] asserts that surgical damage, not the injury, plays a fundamental role in the etiology of knee pain after nailing. Knee pain after intramedullary nailing is most likely related to the operative procedure in the nail insertion rather than the traumatic event which caused the injury.

## Conclusion

Knee pain is a common complication of tibial shaft fractures treated with intramedullary nailing. A significant cause of knee pain appears to be violation of the proximal tibiofibular join by oblique locking screws. The surgeon should be careful not to penetrate tibiofibular joint when utilizing this nail design.

## Consent Statement

Written informed consent to publish the case reports and the accompanying images was obtained from all of the patients concerned.

## Competing interests

The authors declare that they have no competing interests.

## Authors' contributions

PJL and JSF were responsible for developing the idea of this study. PJL, JSF, RESP, HJAF and FBR contributed to the study design. PJL was involved in reviewing records and in data acquisition. PJL, JSF, HJAF, RESP, FBR performed the literature review and drafting of the manuscript. PJL performed all surgeries.

All authors were involved in reviewing and editing the manuscript and all have approved the final manuscript.
